# Nutritional Influences on the Health of Women and Children in Cabo Delgado, Mozambique: A Qualitative Study

**DOI:** 10.3390/ijerph17176205

**Published:** 2020-08-27

**Authors:** Adelaide Lusambili, Violet Naanyu, Gibson Manda, Lindsay Mossman, Stefania Wisofschi, Rachel Pell, Sofia Jadavji, Jerim Obure, Marleen Temmerman

**Affiliations:** 1 Centre of Excellence in Women and Child Health, Aga Khan University, Nairobi P.O. Box 30270-00100, Kenya; vnaanyu@gmail.com (V.N.); stefania.wisofschi@aku.edu (S.W.); jerobure2000@gmail.com (J.O.); marleen.temmerman@aku.edu (M.T.); 2 Department of Population Health, Aga Khan University, Nairobi P.O. Box 30270-00100, Kenya; 3 Aga Khan Foundation Mozambique, Maputo P.O. Box 746, Mozambique; gibson.manda@akdn.org; 4 Aga Khan Foundation, Ottawa, ON K1N 1K6, Canada; Lindsay@akfc.ca (L.M.); rachel@akfc.ca (R.P.); Sofia@akfc.ca (S.J.)

**Keywords:** mothers, women, breastfeeding, infants, children, malnutrition, undernutrition, stunting and Mozambique

## Abstract

In 2017, the Government of Mozambique declared localized acute malnutrition crises in a range of districts across Mozambique including Cabo Delgado. This is in spite of intensive efforts by different non-governmental organizations (NGO) and the Government of Mozambique to expand access to information on good nutritional practices as well as promote nutrition-specific interventions, such as cooking demonstrations, home gardens and the distribution of micronutrient powder to children. This paper examines and discusses key nutritional influences on the health of pregnant and breastfeeding mothers in Cabo Delgado province, Mozambique. We conducted 21 key informant interviews (KIIs) with a wide range of stakeholders and 16 in-depth interviews (IDIs) with women. In addition, we conducted four focus group discussions with each of the following groups: (1) pregnant adolescent girls, (2) pregnant women >20 yrs, (3) women >20 yrs with babies <6 mths who were not practicing exclusive breastfeeding, (4) women >20 yrs of children <2 yrs and (5) with fathers of children <2 yrs. Data were analyzed thematically using NVIVO software. There is no single widely held influence on pregnant and breast-feeding women’s nutritional decision-making, choices and food consumption. Rather, variables such as social-cultural, environmental, economic, gender, knowledge and information intersect in their roles in nutritional food choices.

## 1. Background Information

Malnutrition is a global public health concern and every country of the world has a malnutrition problem irrespective of its wealth [1]. Globally, evidence indicates that 1 in 5 children are stunted and 15.9 million are stunted and wasted [1,2,3,4,5]. Evidence also shows that diets of children and infants are sub-optimal in both high and low-income countries [3,4]. In addition, many countries have geographical areas where the majority of the populations cannot afford nutritious diets [2]. Overall, an estimated 11% of total disease burden is directly attributed to poor nutrition among pregnant women, mothers and their children [2]. 

Good nutrition is foundational to maternal and child health [1,2,3,4,5]. A plethora of evidence shows that poor nutritional status of women affects both their health and that of their children [6,7,8,9]. For example, disabilities, high rates of child morbidity and mortality, stunted growth, diminished cognitive abilities, low educational achievement and disease predispositions in adulthood are all associated with poor maternal nutrition [1,2,3,4,6,7,8,10,11]. In addition, adverse birth outcomes of maternal malnutrition such as low birth weight and preterm birth increase the risks of neonatal death and long-term disability in low and middle-income countries [LMICs] [11,12,13,14].

Nutritional studies in LMICs illustrate that most women lack awareness of appropriate diets and knowledge of food groups and nutrient sources, and few follow a balanced diet [15,16,17,18,19,20,21]. Further evidence suggests that poverty, compounded by social, cultural and religious beliefs may influence nutritional intake for pregnant women [17,18,20]. For instance, women in LMICs are likely to be unaware of the impact of poor nutrition during pregnancy and the first 12 months post-partum [2,4,5,8,18,20]. Equally, religious beliefs, such as fasting, may obstruct women’s food intake and affect the health of women during pregnancy [22]. Similarly, there is burgeoning evidence that links cultural aspects of food to associated food taboos [19,20,21,22]. For example, in some cultures, pregnant women are likely to feed their husbands and children first even when food is scarce, and a boy child is likely to receive more nutritious food and be fed first compared to a girl child [21]. Extreme food restrictions on pregnant women and children, especially food that contains high proteins and vegetables that are green in color, have been reported in literature as they are deemed unhealthy in some cultures [16,17,18,20]. Research from Ethiopia, Zambia and Kenya shows that where extreme food taboos exist, young, poor and illiterate women with low socio-economic status are likely to follow these strictures [15,16,19,22]. 

Malnutrition is high in Mozambique [23,24,25,26,27]. It is estimated that 1 in 2 children are nutritionally deficient, while 52% of children less than 5 years of age in Cabo Delgado are moderately or severely stunted due to low rates of exclusive breastfeeding, limited vitamins, low consumption of iodized salt and high rates of anemia among pregnant women [23,24,25,27]. Research shows that some of these factors coupled with a predominantly starch-based diet have left undernutrition among children under 5 years at 44% [23]. Malnutrition is amplified by food insecurity [24,25,28]. Approximately 23% of the population experiences chronic food insecurity as agricultural production is variable and frequently compromised by natural disasters [26,29,30]. 

In early 2017, the Government of Mozambique declared localized acute malnutrition crises in a range of districts across Mozambique, including the Cabo Delgado province. This is despite intensive efforts by different non-governmental organizations ([NGO)] and the Government of Mozambique to expand access to information on good nutritional practices as well as promote nutrition-specific interventions, such as cooking demonstrations, home gardens and the distribution of micronutrient powder to children less than 2 years of age. It is upon this backdrop that we conducted this research to understand how socio-cultural factors, including those linked to gender dynamics, might influence nutrition practices for pregnant women, breastfeeding mothers and their children. In the following section, we will discuss the methods used in this study.

## 2. Methodology

**Social cultural context**: The study was conducted in Cabo Delgado province with a representation from coastal districts (Quissanga and Metuge) and inland districts (Meluco and Macomia). These districts are within the context of Access to Quality Care through Extending and Strengthening Health Systems (AQCESS) programming and where Aga Khan Foundation Mozambique (AKFM) operates. Compared to National levels, poverty is high in the province. The province scores low on social indicators such as education, health and institutional capacity. Cabo Delgado has rich natural resources such as forest, fisheries, minerals and rich agricultural soils. Although some parts of the province are vulnerable to flooding, the extensive coastline and rich soils provide a basis for fisheries and subsistence farming. Due to the availability of natural resources like forests, hardwood logging for export is widely practiced despite government restrictions on the exports of logs [31].

Study design: The study employed complementing qualitative mixed methods approaches including Key Informant Interviews (KIIs), In-Depth Individual Interviews (IDIs) and Focus Group Discussions (FGDs) (Appendix B). 

**Study sites:** The research sites were diverse and included both coastal and inland sites with easy and poor access to amenities (e.g., roads, hospitals and markets/district hubs). Sites with a variation in socio-cultural makeup and agro-ecological conditions (e.g., high and low potential areas for vegetable production) were included. In addition, places that have both the presence and absence of community nutrition groups as well as areas with the presence of NGO and Government-focused nutrition interventions were included. The purpose was to have diverse study sites to provide a more fulsome picture of nutrition related issues in the targeted areas. 

**Study Population and sampling methods**: To have a broader understanding of the nutritional attitudes, local norms and how women make decisions about food choices, study participants were drawn from a diverse population. Women and men with children, and diverse cadres of community level informants including community leaders, senior men and women, community nutritional groups, village development organizations, community health workers, gender and youth focal point leads were approached and interviewed across the four study sites. 

We conducted FGDs with pregnant adolescent women, pregnant women >20 yrs, breastfeeding/non-breastfeeding mothers with babies <6 mths, mothers >20 yrs with children <2 yrs and fathers with children <2 yrs. The FGDs explored factors influencing nutritional choices as well as the role of gender dynamics in nutritional options for mother and child and infant-feeding practices. Perceptions around current and past nutritional interventions and how they were interpreted and acted upon in light of the prevailing nutritional attitudes and value systems were explored and discussed.

We conducted individual In-depth Interviews (IDIs) with pregnant women and mothers. IDIs were used to explore socio-cultural, gender and economic factors that influence nutrition of women and children. To gain insights into current and past efforts at policymaking around food and nutrition and the influence of these policies in supporting improved access to equitable distribution of food resources for women and children, key informant interviews were also conducted with government officials in policy implementation at different levels (Appendix B.2).

The FGDs were composed of 8–10 participants, and they were purposively identified by recruitment liaisons such as Community Health Workers (CHWs) and/or local level leaders. 

Four women at each site were selected to participate in IDIs. Interviewees were drawn from the population of pregnant and breastfeeding women, including adolescents, as well as the population of women with children less than two years of age. Identification of participants for the IDIs was purposive and with the assistance of the Key Informants (KIs) (Appendix B.1).

Four KIs were engaged per site and were drawn from community leaders/elders, CHWs, leaders of community groups and youth/gender focal points. They were purposively identified as able to provide rich contextual information. KIs from national, provincial and district government were also purposively selected. Table 1 below shows a summary of the study population.

**Interview process:** Interviews commenced after explaining the study and receiving informed consent from the participants. The information obtained from KIIs, IDIs and FGDs was audio recorded with the permission from the respondents. Both IDIs and FGDs lasted no more than one hour. FGDs were conducted in spaces and places that were chosen and deemed convenient and private by the participants. Facilitation of FGDs sessions were gender sensitive (females facilitated females FGDs and similarly males facilitated male FGDs sessions). Following the completion of the interview or discussion, the audio recording was backed up securely and encrypted by the Monitoring and Evaluation Research Learning (MERL) unit at the Aga Khan University. Researchers conducted daily routine debriefs that entailed sharing of significant findings, personal reflections and any key interpretations of findings that required further probing. For example, a typical debrief entailed listening to at least one interview, ascertaining the quality of the information in terms of the process and translation from local language to Portuguese to English.

**Ethical Considerations:** The study protocol was approved by Mozambique Ministry of Health, bio-ethics committee # IRB00002657. Endorsement for the study and permission to carry out fieldwork in study sites was sought from the leaders of each community where the study was conducted. Prior to the start of any data collection activity, the objectives of the research were explained to participants in their own language allowing them to confirm that they have understood, and that participation was voluntary. Informed consent (and assent for minors) was sought from each participant—both verbally and in writing (Appendix A). 

## 3. Data Analysis

All interviews were transcribed and translated from local languages to Portuguese and then to English. The lead local consultants oversaw the work of the nine transcribers and translators and ensured that quality translations were produced by randomly checking the scripts. All transcripts were coded, and the data organized and analyzed with the use of NVIVO software. The study followed six phases of thematic analysis as described by Braun and Clarke (Braun, 2006): (1) becoming familiar with data, (2) generating initial codes across the data set and grouping coded data, (3) searching for themes by collating analysis codes into possible themes and gathering data that is relevant to each possible theme, (4) reviewing themes and creating a “map” of the analysis, (5) defining and naming themes and (6) producing an analysis report and selecting appropriate, vivid quotes in support of described themes. Table 2 below illustrates the themes that emerged.

## 4. Findings

Across the four rural study sites where the research was conducted, pregnant and lactating women’s decisions about what food to buy, eat and feed their young children were affected by culture (local traditions and mores, religious strictures, personal preferences), logistics (food availability, affordability), economics (access to resources to buy food) and social status (education level, knowledge about childhood nutrition). Findings suggest that, although certain food taboos for pregnant and breastfeeding women and infant children exist, no beliefs about foods as appropriate or inappropriate were widely held across the study region. Foods considered bad or forbidden at one research site were readily consumed by women in other areas of the same district, suggesting that food taboos were highly localized rather than the result of a generalized system of beliefs. The following themes summarize nutritional influences on the health of women and young children in Cabo Delgado, as gleaned from interviews conducted during this study.

1. Lack of food diversity and harmful weaning customs;
2. Eat a pig and give birth to a pig: Intersection between lack of knowledge and social and cultural influences on food consumption;
3. Food choices determined by accessibility;
4. Economic influences on food choices and consumption;
5. Differential gendered influence on food consumption and allocation.

The findings below, largely from FGDs and KIIs, represent common patterns in participant’s views across the study sample. 

### 4.1. Lack of Food Diversity and Harmful Weaning Customs

Many participants reported very little variation in the kinds of nutritious foods available for infants and young children. Interviews further suggested that few mothers were aware of children’s nutritional requirements or of how local food habits and traditions affected what they fed their children. Across the four study sites, findings showed an overreliance on starches (such as *xima* made from maize meal, cassava, sorghum flour and plain porridge), with few nutritious foods (such as vegetables, eggs, meats and fish). When asked about what they feed children younger than 2 years old, findings from FGDs suggested that children below two years ate the same foods as adults (such as porridge and “*mutranca*”), in large part because their mothers had no alternatives.

*We cook and we all eat the same food. There is no food for children or for adults. We all eat the same food. (Meluco, FGD, Mothers with babies under 6 months, 20–40 yrs.)*

*…There are no other foods to give these children we give to them what we have to eat…. (Macomia, FGD, Mothers with children under 2 years, 20–39 yrs.)*

*… I give the child this sort of food, because that is the food I have. If I have cassava pap, I prepare it to eat with my son…. (Quissanga, FGD, Adult pregnant woman, 24–36 yrs.)*

These statements align with those expressed by both key informants and IDI participants.

*We generally give them “muatranca”. It does not matter if one is 1 year old or 2 years old. We do not have a choice. (Macomia, KII -AG-1).*

*Here we eat cassava porridge and maize porridge. This is the food we are used to… Every year the same food and because we produce the same food, we cook the same food all the time, and the child eats this same food and grows. (Meluco, IDI, Pregnant adolescent, 19 yrs.)*

*We give them porridge, cassava dough that is why they get weak and do not develop because they are not yet old enough to eat, they must be at least six months old. (Macomia, IDI, breastfeeding women).*

In addition to a lack of food diversity, study findings, as described by women, illustrate some measures taken to wean their young children. These measures include giving young children cookies and putting pepper or adhesive on their breasts. These practices are likely to be based on societal customs to incentivize children to wean. However, women may not take into account nutritional considerations when applying these practices. The following excerpts by different pregnant women from an FGD in Macomia provide further insights into local customs surrounding the weaning process:

*Respondent 1: When I wean the child, I buy refreshment with cookies for him/her. When he/she asks for me to breastfeed him/her, I just give this to him/her, in the evenings too. So, when she eats, she forgets about the breast milk. (Macomia, FGD, Pregnant Adult Women, 24–36 yrs.)*

*Respondent 2: They take adhesive and stick on the breast for the child to be afraid. They also put red flowers around and lie that is blood and the child stops nursing because he is afraid of these things the mother has put. (Macomia, FGD, Pregnant Adult Women, 24–36 yrs.)*

*Respondent 3: Others come to the point of rubbing piripiri [pepper] for the child to feel bitter/hot and think that is dangerous and then the child gives up. (Macomia, FGD, Pregnant Adult Women, 24–36 yrs.)*

These local attitudes and practices surrounding weaning bring up the question of what does constitute appropriate food for young children.

### 4.2. Eat a Pig and Give Birth to a Pig: The Intersection Between Lack of Knowledge and Social Cultural Influences on Food Consumption

Findings from this study indicate that women and young children in Cabo Delgado primarily eat meals made of maize flour, mainly *xima* and porridge, occasionally supplemented with cassava. 

Breastfeeding women reported preferring porridge, which they believed helped them to produce more milk and to make their children play well. Government officials, who were key informants for this study, emphasized that maize meal (*xima*) and porridge meal both have little nutritional value and are not sufficient for pregnant or nursing women. They reported that maize meal has been the staple diet from generation to generation, and that the preparation and consumption of *xima* and porridge is culturally embedded in families’ routines. As highlighted by a senior government official, these nutritional traditions may conflict with modern medical ideas about the kinds of food pregnant and lactating women require.

*…The family says she should not eat this or whatever; I have been to a province, in which, to consume the flour made in Tete, it has to be put into water for two up to 3 days and grind. All nutrients disappear. Let me tell you this. Maize flour is delicious. I love that, but it has no nutrients. …. they prefer that flour due to the food habits. They eat that flour with dry fish. For them, to mix greens, it means to break the flavour. Those are the food habits which people have, and they disseminate it to all generations, consequently it gets difficult. **…** it is not just in the rural communities, but also in the urban ones. There is lack of nutrition understanding. We have to know eating a lot does not mean to eat well. … They prefer not to take vegetables, various greens which are nutritional (Maputo, Key Informant, Government official)*

A senior nutritionist who was a key informant for this study explained that people in rural areas had little knowledge about nutrition and generally held on to traditions that interfered with women’s ability to make nutritious food choices. In the following interview, the dilemma faced regarding food choice for pregnant women in rural Mozambique is explained.

*Interviewer: …what do you think are the main difficulties affecting access to nutritious foods for pregnant women, mothers and children under 2 in rural communities?*

*Respondent: The first difficulty is lack of knowledge concerning what these nutritious foods should be; the second difficulty is the acquisition of products.*

*Interviewer: What are the difficulties for pregnant women and infants under 2 years old to adopt good nutritional practices?*

*Respondent: One of the difficulties is the myths and beliefs people have: pregnant woman must not eat this, child must not eat this, and this makes pregnant woman lose some essential nutrients and then poor nutrition of pregnancy begins. Besides this, they introduce food very early. They start to give drinks very early because they say that the child is thirsty. In short, these myths are the matters that lead to bad practices and not only but also the lack of knowledge, but if we convey the information that this is right and that is wrong, and I do believe we can reduce the issue of myths and beliefs in communities. (Pemba, Key Informant, Nutritionist)*

As the above interviews show, preferred traditional food preparation procedures may be compromising food’s nutritional value. Similarly, as demonstrated above—and in interviews with other government officials and policymakers—people in our research sites may lack understanding of nutritional values in foods and are reluctant to break with longstanding food-related traditions. 

Local food taboos as illustrated in Table 3 further complicate food choices for pregnant women and children. Excerpts from FGDs and KIIs suggest, in addition to an almost complete lack of knowledge about food value, rural Mozambiquans tend to mistrust foods, especially proteins outside their maize-based local diets. To some participants, eating rice and fish was considered a sign of being poor, while eating starches such as cassava and maize meal was associated with happiness.

*The healthy food that provides health to people is “Celeste” pap with cassava leaves. We eat rice with fish for being poor, for not having a choice; it is unhealthy food. (Quissanga, FGD, Fathers with children under 2 years, 20–45 yrs.)*

*The food that strengthens is “sorghum”, cassava leaves with peanuts and beans. This food does not only strengthen, but it also increases blood. (Quissanga, FGD, Mothers with children under 2 years, 20–31 yrs.)*

*… our food is Xima, cassava Xima, we buy at 200 mts, and we clean it and take it for grinding and we find vitamins. (Metuge, FGD, Pregnant adolescent women, 15–19 yrs.)*

*Here the food that we rely on a lot and we eat with our heart is only porridge from maize. When we eat Xima [porridge] we feel happy and the body feels good. But when we eat rice and xima of cassava, we only eat for the sake of eating only. That maize is what we like the most. (Macomia, KII, Male community leader)*

The ability to change the communities’ views and behaviours on what constitutes appropriate food to consume while pregnant was hampered by existing taboos. For example, taboos on which foods to eat while pregnant, breastfeeding or for a young child existed across the districts but were not homogeneously held by all the people as some practiced them while others did not. A government official below illustrates this,

*In some places, there are myths. For example, you must not eat an egg, otherwise, you will give birth to a bald child among others. It has to do with the areas; food habits in the coast area. People are used to eating fishes with cassava flour while it is bad for them, because it has no nutrients, especially for pregnant women. It ends up affecting the babies from 0 up to 24 months old. (Pemba, IDI, a government official)*

As seen in Table 3, prohibited food are mainly proteins, and avoiding them could compromise the health of pregnant and lactating women and developing children. Participants across the study sites reported that elders in their community transmitted information regarding these taboos from generation to generation.

*These ideas come from the elders of our community. They know about the harms caused by those habits in the past and… (Macomia, KII, Male, Community Health activist)*

*Elders are the ones who tell us. After you are pregnant, elders let you know, ‘You are pregnant, you ought not to eat things like this.’ They tell you. You do not eat, because you have been warned. (Macomia, FGD, Pregnant adolescents, 15–19 yrs.)*

*Usually the elders are the ones who tell us what a pregnant woman does not eat because they have seen these things happen, and if a person has ears to listen, she won’t eat, but if she insists that they are old ideas and eats, what the elders said would happen does happen. (Meluco FGD, Pregnant adolescents, 15–19 yrs.)*

As illustrated in the following quotes from the FGDs, where there is information on the right foods to be eaten, people may not understand or are reluctant to apply what they have learned, which may explain the region’s overreliance on starches.

*We eat any food, whatever comes along because no one has ever told us that we should eat this food when you have a child who is 6 months or under. (Macomia FGD, Mothers with babies under 6 months, 20–40 yrs.)*

*Some do, but like I said earlier not everyone understands. Therefore, we go little by little. People will not understand you at once, on the first time. We have a health agent, every Friday and Monday holds lectures to explain to people that they have to eat this, … this, to have energy. On the days we hold meeting here in the community, we explain to women who are pregnant, those who are breastfeeding that they should eat this, this, this, this, which I just mentioned. (Macomia, KII, Male, Community nutrition group leader)*

As it can be seen from these interviews, there is a general preference for starchy foods compounded by a lack of information and knowledge, taboos and misconceptions on what constitutes appropriate foods and what does not. 

### 4.3. Food Choices Determined by Accessibility 

Women reported that accessibility was the major factor determining which foods they ate. Rural participants seemed to feel that so long as food was in season—such as cassavas or maize meal—it was nutritious. According to their explanations, cassavas and maize meal provided more energy than vegetables and were better for building up the body.

*We get food such as “sorghum”, “Celeste” flour even “nhemba” beans every year. Papayas, bananas and other food are hard to find. (Quissanga FGD, Mothers with children under 2 years, 20–31 yrs.)*

*I have not eaten anything since morning and, at home, there is not pap for porridge, only cassava pap. My sons/daughters’ father is absent who could go work to bring maize pap. We eat cassava pap that we cultivate. This year, there is no rice, not even maize, only cassava. So, I breastfeed and my heart aches. I cry. (Metuge FGD, Mothers with children under 2 years, 23–43 yrs.)*

More women reported that they ate what was accessible and considered it nutritious.

*The nutritious and energetic food is the paste of pounded cassava in its majority, maize flour, sorghum, but rice is not frequent, these are the only foods we use. (Meluco, KII, Female, Community group leader)*

*It must be maize porridge because it gives strength; oil builds the body, with beans leaves because they build the body. (Macomia, FGD, Mothers with babies under 6 months, 20–40 yrs)*

*For food with vitamins we have cassava leaves, mooring with peanuts, oil is nothing but with peanuts, onion, salt with cornmeal/porridge. After a while you prepare lettuce and eat, if you have an orange, banana, cashew you can eat. (Quissanga, FGD, Pregnant adolescents, 15–19 yrs.)*

*Food that will give you strength is maize with cassava leaves because and to diversify we when we put aside muantopa [porridge made of cassava flour] we eat this to have strength. (Meluco, FGD, Mothers with children under 2 years, 20–44 yrs.)*

These views highlight a lack of food diversity and the need to introduce more diverse and nutritious crops.

### 4.4. Economic Influences on Food Choices and Consumptions

Participants reported that while food availability was a key determinant of what they ate, economic factors also played a role. Participants observed that foods found in exclusive shops, such as rice, spaghetti, and fish, were consumed by those considered wealthy (mostly, in this case, people with government jobs). Some women and young adolescents reported living in economic conditions so dire, they could barely purchase food, no less food they considered nutritious. Even participants with small household businesses (such as cutting wood, fishing, and selling food in the informal sector) were too poor to spend extra money on food.

*Our husbands go fishing. The day they get fish, they get no clients unless people from Pemba or Montepuez come to purchase them. The day he does not succeed in selling, he puts the fishes to dry so that he can eat with the family. (Quissanga, FGD, Mothers with babies under 6 months, 20–36 yrs.)*

*Selling food is difficult because those who come to buy fix very low prices that do not befit us and we end up losing. (Meluco, FGD, Fathers with children under 2 years, 28–45 yrs.)*

*There is a shortage of money here in the village; you can make cakes to sell but no one buys. (Metuge IDI, Adult Pregnant woman, 34 yrs.)*

*Young people cut wood, but their wood takes a long time to sell. Here it is difficult to have money, even when making cakes; you do not finish [selling them] because there is no money in this community. (Meluco, KII, Female, Nutritional leader, 28 yrs.)*

Below are some snippets from an FGD with pregnant adult women in Macomia, that provides insight on how financial factors impact women’s decision making.

*Respondent 8: It depends on the financial condition of each person. Some eat rice; others eat cassava porridge, because they cannot afford other products. This is what makes the difference, others can only eat porridge while others eat meat.*

*Respondent 1: There are differences on eating habits because of the financial conditions; others can have cassava leaves accompanied with cassava porridge. Those who are blessed by God eat rice with fish, while you eat cassava leaves, that is the destiny [suffering] God gave you.*

*Respondent 5: People have different fortunes. Some will eat cassava vegetables with cassava porridge, but we also would like to eat rice, but it is difficult to have it. (Macomia, FGD, Pregnant Adult Woman)*

Individuals who are poor also ate nutritious food such as sorghum and cassava; however, by and large, they lacked diversity and some of them did not perceive these foods as nutritious. Those with more wealth have more options of food to buy.

*…This in Makonde is called poverty. If you do not have money you will have difficulty to eat, you will eat poor food. But those who have money eat the food they want because money allows you to eat well at home…those with money do not eat cassava, sorghum. People with money always eat rice…eating well differs because of not having money. (Macomia, KII, Male, Community leader)*

*First the food rich people—those who have money—eat, which we, the poor cannot afford because we don’t have money is manufactured or industrialized food because it is very difficult to find it like the case of spaghetti… things you may feel like eating but either you can’t find or afford them, like milk, for example. (Macomia, KII, Male, Community nutrition group leader)*

*We eat cassava pap, because we do not have maize pap. Who gets money, purchase maize pap that costs 1500 Meticais to 2000 Meticais each cup or rice which costs more. It depends on your money, if it is a lot or little. If you do not have, cook cassava pap. (Metuge FGD, Mothers with children under 2 years, 23–43 yrs.)*

*There is rice, macaroni, sardines, oil, and spice. When they start cooking, it smells pleasantly and we, who do not have it… our cassava leaves will not be all right even though we will take it and cook with “muatranca”…. (Meluco FGD, Mothers with children under 2 years, 20–44 yrs.)*

In this theme, we see that differential socio-economic status, as would be expected in each society, influences how different people make decisions and choices on food consumption.

### 4.5. Differential Gendered Influence on Food Consumption and Allocation

Across the four research sites, gender and social norms were not key determinants of food choice; however, findings suggest that they were relevant for food consumption and food allocation. Moreover, both men and women decided what food to buy. It was reported that men who worked had money and contributed to the household food purchasing. Some households distributed food on the basis of gender, with women in FGDs reporting eating separately after cooking. In some instances, food portions were based on the availability of food within the household and influenced by the number of family members and their ages.

*There is no difference; they all eat the same quantity. The thing is their mother considers a certain quantity of porridge to be sufficient and that the children will feel satisfied. (Quissanga FGD, Mothers with children under 2 years, 20–31 yrs.)*

*I do not separate because in this village we do not usually give each child their plate. I have 4 children; they all eat in the same dish. Only the father has in his plate. I and my children eat together. (Meluco FGD, Pregnant adolescents, 15–19 yrs.)*

*There is no difference, they both eat the same food. If we are to eat bran both male and female children we eat bran. (Macomia FGD, Fathers with children under 2 years, 20–31 yrs.)*

*Each child eats taking into account the age. I divide like this: I serve two serving spoons for the 5-years-old child and 1 serving spoon for the 2-years-old child. I mean, it depends on the ages. The amount of food that I serve for an adult person differs from the one I serve for 5-years-old child. (Quissanga FGD, Mothers with children under 2 years, 20–31 yrs.)*

*It is not divided equally. It depends on each one’s age. Children have a different food amount from elders. (Macomia KII, Male, Community leader]*

In some places, as revealed in this study, women observed that they served men or their husbands first, stating that this was because men bought the food and cultivated the farms. Women narratives also suggested that men may get the bigger and/or better portions.

*Yes, I did. He is the one who buys the food. There are men that do not allow children to be served first. (Quissanga FGD, Mothers with children under 2 years, 20–31 yrs.)*

*We give the best part for men because they are who cultivate so we put them before women…We give much cassava dough to men and we get the less part because they say men cultivate more, but we cultivate too. (Macomia FGD, Mothers with children under 2 years, 20–39 yrs.)*

*I commence serving my husband and I seat with my sons/daughters and we eat together. I always start with my husband. (Metuge FGD, Mothers with children under 2 years, 23–43 yrs.)*

*When preparing food should start with the father, but because the father may be away, I cook the food inside, and I serve the children to eat. (Meluco FGD, Pregnant adolescents, 15–19 yrs.)*

*…In principle priority is given to the man. You serve him water, food, and he is the first to eat. After serving the man the woman eats. Normally in this village the man is the first. (Meluco FGD, Fathers with children under 2 years, 28–45 yrs.)*

*…Men are the first eating; women cook and when they finish, they call for the boys to take food to the men. Women are always the last ones eating. (Macomia KII, youth, 22 yrs.)*

At all four study sites, some participants reported that both men and women contributed in the process of buying food, and they both worked as women frequently do the unremunerated work of cultivating and managing the home. Men provided finances to women to buy food and sometimes participated in the decisions, but a large number of women made the decisions on what food to buy.

*There are men who when they get money, they buy cassava leaves, oil, and give them to the woman and eat. (Meluco FGD, Mothers with babies under 6 months, 20–40 yrs.)*

*There are men who do not want to be asked; they give you money to go buy what you want, and he eats without queries. (Metuge, FGD, Mothers with babies under 6 months, 20–27 yrs.)*

*It is the woman who makes the decisions…The woman knows what is missing at home. (Quissanga, KII, Male, Nutrition focal point, 45 yrs.)*

*Women are the ones who make this decision [to purchase food] because they are the ones who cook. (Macomia, KII, Male, Youth/Gender focal Point)*

In most of the communities, as seen from the above narratives, although men are the ones providing finances, women are frequently making decisions about what to purchase and cook. Further, the food prepared in the household is for the most part shared among all household members, although in some cases men are given bigger portions of foods. In some places, allocation of portions of a meal primarily began with men and elders first, including guests of the male head of the household and portion sizes decreased concurrently with age, with children receiving smaller portions given their age and size. 

## 5. Discussion

**Figure 1:**
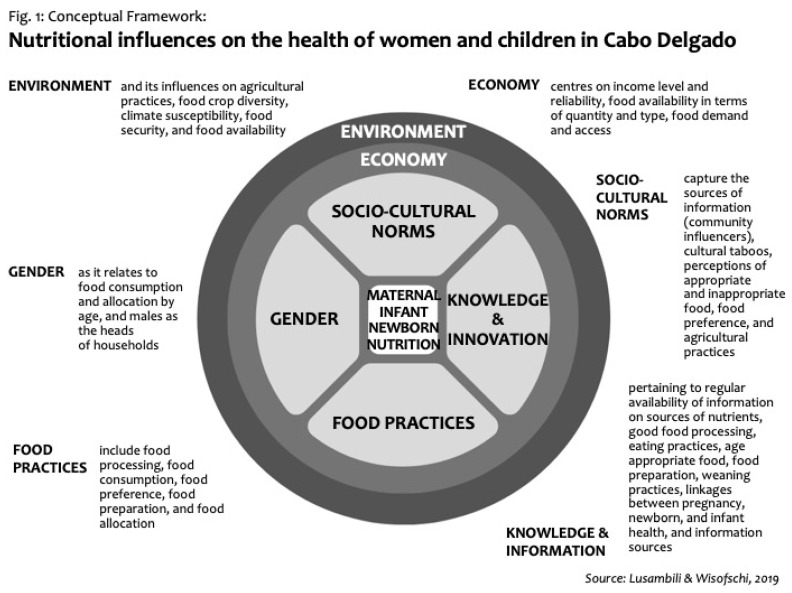
Conceptual Framework.

This paper aimed to examine and present some of the nutrition related influences on the health of pregnant and breastfeeding women, infants and developing children in Cabo Delgado province, Mozambique. The findings presented in this paper are from FGDs, IDIs and KIIs with a wide range of women, both pregnant and breastfeeding, community leaders, fathers and government officials. Below (Figure 1) we present a conceptual framework that summarizes the findings of this study.

As shown in the above figure, a wide range of variables may influence pregnant women’s decision in buying and consuming food. The staple diet consumed by women and children in Cabo Delgado consists of *xima* (made of maize meal), porridge and, occasionally, cassava. These foods are all starches and provide none of the proteins, vitamins and minerals required for women and developing children to thrive. These choices must be understood within Cabo Delgado’s social, cultural and environmental context, which may dictate what foods are grown and consumed. Climatic seasonality may dictate food availability as well as food diversity and security, whilst social and cultural norms may have a bearing on what foods are preferred and thus planted. Further, cultural influencers, such as elders, reinforce some of the food taboos, which may direct what information and knowledge women have concerning appropriate and inappropriate foods. In addition, social and cultural beliefs are powerful in dictating food processing (drying fish and soaking maize meal in the water remove nutrients) and food consumption. Economic influences were also identified as important in determining food consumption. Many of the Cabo Delgado residents interviewed cannot afford sufficient food to feed the family; most participants have no access to foods outside the traditional diet and could not afford them even if they were available. The health benefits of proper nutrition are generally unknown throughout the District. Participants report methods of food preparation that remove nutrients from foods. Local food taboos reinforce the preference for the traditional maize-based diet. These findings are consistent with findings from similar research in LMICs, for example, gender and food consumption [20,21], taboos about proteins and green vegetables [16,18,19,20], lack of knowledge, information and education [15,16,17] and environmental factors [24,28,30]. Contrary to the existing literature from LMICs [21,22], findings do not suggest that boys are fed first or given nutritious foods compared to girls. However, findings also suggest that men were fed first as part of the household arrangement.

## 6. Proposed Recommendations

As it can be seen from these findings, the general preference for starchy foods and lack of information on how to preserve nutrients during food preparation is compounded by local food taboos. These views may suggest moves to increase diversity in the rural diet, which must be accompanied by outreach to inform local communities about nutritional requirements and healthy preparation methods. To make sure the information is accepted, members of many different subgroups within the local society should be involved in its dissemination. Women and men should be given information on the benefits of diversifying the diets of children and women particularly during pregnancy and breastfeeding and the importance of not feeding their children non-nutritious foods. 

There is a need to develop a holistic strategy aimed at the communities including health care providers, CHWs, fathers, mothers, elders and adolescents on appropriate food habits. This research found that people are getting the information but are still not changing practice and demonstrating why a different and/or more holistic approach is required.

Further, in communities where harmful beliefs and taboos are strongly practiced, there is a need to educate the women and work with the elders so that they do not restrict their diet when pregnant or breastfeeding as well as the community influencers [elders] who reinforce these norms. For harmful weaning behaviors, mothers and communities at large should be educated on the adverse effects of putting adhesive material or pepper on women’s breasts, or feeding children processed foods such as sodas, juices, cookies and cakes that contain added sugar. There is need to focus more information on healthy foods that will improve health outcomes for pregnant and lactating women, infants and developing children. 

Similarly, the government of Mozambique must put strategies in place to ensure that there is relevant information about nutritious foods and ways to prepare food to retain nutrients. Information about the nutrition needs of pregnant and lactating women and small children must be increased, and there should be measures to engage community influencers, women, men and others on these topics and to start to shift norms around taboo foods and common practices around weaning. In addition, larger scale approaches to address availability of nutritious food at an affordable rate, mechanisms to address household and community poverty rates and improve food diversity should be implemented by the government.

Lastly interventions targeting influencers such as the elders who promote taboos that prohibit pregnant, breastfeeding and young children from eating animal proteins should be encouraged through media and other social avenues. 

## 7. Proposed Research

These findings suggest the need to conduct evaluation research on interventions that have been effective to shift norms around nutrition for pregnant and lactating women in Mozambique. Such research would provide insights on what has worked around addressing some of the issues related to food beliefs and practices. Mixed methods research would also provide disaggregated information about the effects of these food practices on various populations. Of interest will be research to establish if there is a significant or/and any difference in stunting and wasting between a girl child and a boy child. Further, qualitative research is needed to shed light on how the population of Cabo Delgado understands the etiology of illnesses and diseases in the context of the above influences.

Since the variables that influence women food choices and consumptions are varied, it will be insightful to understand quantitatively which variables have the greatest effect and what could be easily modified. To have a better illustration of how variables such as social-economic status influence maternal food choices and therefore nutritional outcomes of children, quantitative research examining a multiple factor analysis of local Gross Domestic Product (GDP) per capita and Engle’s co-efficient of the interview household and health status of the child(ren) should be considered. In addition, quantitative research could examine the influence education and religious background have on food selection and nutrition of pregnant women. Lastly, it would be important if future research could look at the extent to which household size and early motherhood and breastfeeding practices affect the prevalence of malnutrition in children.

## 8. Conclusions

Using complementing qualitative mixed methods, this study aimed to gain insights on how socio-cultural factors, including those linked to gender dynamics, might influence nutrition practices for pregnant women, breastfeeding mothers and their children. The findings show that a wide range of influences such as the intersection of knowledge and social-cultural norms, economics and environmental and gender variables continue to shape how women make choices about food consumptions. The findings have expanded our knowledge on influences of women’s food choices in Cabo Delgado that is crucial for cultural and tailored policy implementation.

## 9. Study Limitations and Strength

The current study was conducted in Cabo Delgado province, and although the findings from this study may have some similarities across Mozambique with respect to factors that influence food consumptions, the findings cannot inform national interventions, as they are limited to one province. In addition, this research was conducted in areas where the Aga Khan Foundation Mozambique (AKFM) is already actively working on nutritional programs. Thus, it is likely that these linkages and familiarity with AKFM may have compromised interviewees’ views during the interviews.

The strength of the study lies in methodological approaches whereas we employed complementing multi-qualitative methods (FGDs, IDIs and KIIs) on a wide range of participants focusing on local people’s lived experiences. Although the findings of this study are consistent with findings in LMICs, the current study provides a rich description of food consumption practices for pregnant women, breastfeeding women and children under 2 years, particularly in selected districts of Cabo Delgado. Compared with previous research findings that have shown influencers to pregnant women food consumption as isolated, this study, as illustrated in our conceptual framework, deepens our understanding on how different variables intersect in women’s food choices and decision making.

## Figures and Tables

**Table 1:** Study population and segmentation by methodology.

Type of Respondent	Sites			
	Ibo	Metuge	Meluco	Macomia
Key informants Including 5 KIs with government officials	4	4	4	4
In-depth interviews with mothers	4	4	4	4
Focus group discussions (8–10 members)				
With pregnant adolescent women	1	1	1	1
With pregnant women >20 yrs	1	1	1	1
With women >20 yrs with babies <6 mths who were not practicing exclusive breastfeeding	1	1	1	1
With women >20 yrs of children <2 yrs	1	1	1	1
With fathers of children <2 yrs	1	1	1	1

**Table 2:** Codes, categories and themes.

CODES	CATEGORIES	THEMES
1. We have no specific food for a child. We share same food with children. We feed our children cassava. Our children are fed maize meal. We feed children cookies and sodas during weaning. We put pepper on our breasts to wean children. We put adhesive stuff on our breasts to wean children.	Limited food choices for children; Starchy foods. Harmful traditions on child weaning.	Lack of food diversity and harmful weaning customs
2. We prefer xima and porridge. Strong myths and beliefs about food preparation. Food preparation influenced by myths. Food consumptions passed on from generation to generation. Starchy food preferred for energy. Myths about protein foods. Eat a pig and give birth to a pig. Eat buffalo and you miscarry your child.	Beliefs, myths, lack of information on healthy food preparation. Lack of knowledge. Intergenerational food habits.	Eat a pig and give birth to a pig: Intersection between lack of knowledge on appropriate food preparation and social cultural influences on food consumption
3. More cassava and sorghum are available. Pounded cassava is nutritious. Maize meal gives energy. Maize porridge is good for strength. Maize meal and cassava are readily available.	Accessibility misconstrued for nutritional food. Lack of knowledge on what constitutes nutritious food.	Food choices determined by accessibility
4. Exclusive food is for rich people. Rice, spaghetti and fish are for rich people. Majority of the population is poor. No customers to buy our fish. No customers to buy our cakes. No customers to buy our wood. Cannot afford fortified flour. Our people are poor. No money to buy unhealthy food.	Poverty. Variation in socio- economic class.	Economic influences on food choices and consumptions
5. Child’s father served food first. Food in the household served based on age. Both women and men make decisions on food choices. Men work and buy food. Men deserve more food.	Age and gender determine food allocation. Patriarchy (men served first).	Differential gendered influence on food consumption and allocation

**Table 3:** List of foods and taboos around food consumption for breastfeeding and pregnant women.

Food	Beliefs
Pig	A child would be born looking like a pig
Octopus	If she ate it, she had to cut its tail, since it caused stomachache, and one could die. A pregnant woman found having eaten it would also be labelled a sorcerer
Buffalo	Causes miscarriage
Shrimp	Causes tummy aches
Sea turtle	A child would bite the mother’s breasts while breast feeding
Meat of an animal that had just given birth	A child would be weak, lack good health A child would have a bad tongue
Animal intestines	They terminate the pregnancy, or the woman would have difficulties during delivery
Eggs	Bald or hairless child
Little bird	A child would have a bad mouth
Coconut	A child would be born “whitish”

## Data Availability

Transcripts from this study cannot be shared publicly due ethical consideration. Researchers who meet the criteria for access to confidential data can contact the following individuals at the Aga Khan University Monitoring and Evaluation Research Unit (MERL): Marleen Temmerman [marleen.temmerman@aku.edu]; Adelaide Lusambili [Adelaide.lusambili@aku.edu]; and research.supportea@aku.edu.

## Abbreviations and Acronyms

AKFC	Aga Khan Foundation Canada
AKFM	Aga Khan Foundation Mozambique
AKU	Aga Khan University
AQCESS	Access to Quality Care through Extending and Strengthening
COEWCH	Center for Excellence in Women and Child Health
FGD	Focus Group Discussion
GoM	Government of Mozambique
ICRH	International Centre for Reproductive Health
IDI	In-depth Interview
INS	Instituto Nacional de Saúde
KI	Key Informant
KII	Key Informant Interviews
NGO	Non Governmental Organisation

## Appendix A. Informed Consent Form

**Part I: Information Sheet** 

**A. Introduction** 

You are invited to participate in a research study on the social, cultural and economic factors that affect the nutrition practices of pregnant women and mothers. You have been selected because you are a resident of this community and are either a key informant who can provide insightful information on the study topic based on your position within the community or you are a pregnant woman of a certain age or a mother/father with a child below two years of age. The information on this form is being provided in order to tell you about the study as well as your rights as a participant in the study. You have the right to ask questions about the study and your involvement. Your participation in the study is also voluntary—you can choose not to be involved in the study or to withdraw your participation even after the study has started and to request that the investigator and assistants conducting the study do not use any of the information you have provided. Doing so will not have any negative consequence for you.

**B. Procedures** 

If you choose to participate in this study, then you will be asked to participate for approximately 90 min. You will be involved in either a face-to-face interview or be invited to participate in a group discussion with between 6-9 other participants who will be of a similar profile to you. All interviews and discussions will be conducted by the investigator and his/her assistants at a quiet venue that is conveniently accessible to you. Prior to the start of the interview or discussion, we will explain the nature of the study and obtain, on this form, your written consent to participate. We will ask you to sign two copies of this form. One copy of the form will remain with you and the other copy with the investigator, who will be responsible for storing the form in a safe place. Once you have provided your consent, then the interview or group discussion can begin.

At the beginning of each interview or discussion, some basic information will be collected from you, including your age, marital status, occupation, education level and primary language. You will be asked a number of questions relating to the types of foods you choose to eat, why you choose these foods, the factors within your household and community that influence your decisions around food—for yourself and your child, as well as some questions around other practices that are important for good nutrition, such as breastfeeding.

**C. Risks and Benefits** 

At no point in this study will you (or your child) be at risk. Your participation in the study should also not cause you any discomfort. However, if you feel uncomfortable with the types of questions you are being asked, you have the right to inform the investigator as well as the right to refuse any question you do not wish to answer, as well as the right to withdraw from the study altogether. You will not receive any payment or other material benefit for your participation. However, you have a non-judgmental opportunity to share your thoughts, beliefs and experiences in relation to the nutrition of mother and child in your community, and the study is intended to be beneficial by allowing for consideration on how to improve wellbeing for mothers and their children in future.

**D. Withdrawal** 

You may withdraw your participation at any time without a need for justifying your reason and without any reprisal or consequence.

**E. Anonymity and Confidentiality** 

Although we will collect your name and signature on this consent form, your name will not appear as part of our analysis of the information you provide. Your name will also not appear in the presentation of results. You will have complete anonymity, and none of the information you provide to us will be directly connected to you in any way. Your personal details will remain confidential, and no one other than the investigator and the assistants will have access to your personal details.

**F. Additional Information** 

At the end of the study, a meeting will be held in Pemba involving representatives from the Aga Khan Foundation, the Aga Khan University and the Provincial Directorate of Health to discuss results. This meeting will also identify how to use the information being collected to improve wellbeing for mothers and their children in Cabo Delgado. While the investigation is underway, the investigator and the assistants are available to answer any question you have. After the investigation is completed in this community, you can still contact Dr. Egas Simbine by telephone (82 718 1060 or 21 490 515).

**Part II: Consent of Participant** 

Having been invited to participate in this study on factors influencing the nutrition of mothers and their children, I declare that:

I have been satisfactorily informed that the purpose of this study is to collect information on the social, cultural and economic factors that affect the nutrition practices of pregnant women and mothers.

I was duly informed about the nature of my participation in the research as well as the risks and benefits that are associated with my participation. I understand that I will not receive any material or monetary reward for participating in the study. I have been duly informed about my right to withdraw from the study at any time without any consequence. I understand that the information regarding my participation will be confidential and that the information I provide will be used by the Aga Khan Foundation for a better consideration of how to improve nutrition of mothers and children, but without any risk to my right of confidentiality.

I also understand that if I have questions, I can contact at any time Dr. Egas Simbine by telephone at 82 718 1060 or 21 490 515 and/or the Comité Nacional de Bioética para a Saúde at 82 406 6350.

I freely volunteer to take part in this study.

*If the participant is illiterate and gives only oral consent and a thumbprint or if the participant is below 18 years of age, then a literate witness must also sign.* 

_____________________   _____________________        __________________

Name of Participant      Signature/Thumbprint of            Date

Participant

_____________________   ______________________        __________________

Name of Witness and      Signature of Witness                        Date

Relationship to Participant

______________________   _______________________        __________________

Name of Investigator      Signature of Investigator            Date

## Appendix B. Study Tools

### Appendix B.1. Question Guide for Key Informant in-Depth Interviews with CHWs, Leaders of Community Groups, Community Elders, Gender and Youth Focal Persons

**Study:** Influence of socio-cultural, gender and economic factors on maternal and child nutrition practices in Cabo Delgado province, Mozambique

**Interviewer number:** _________

**Date:** __________________ **Venue:** _______________________

**Interviewer:** ___________________

**Site/District:** ___________________

**Participant category:** □ CHW

□ Community Elder

□ Leader of specific community group/s

□ Gender focal person

□ Youth focal person

***Obtain informed consent from every participant before proceeding to the introduction and collection of participant data.***  

***KII participant characteristics*** [Collect these details for every KI]

1. Age: ___________

2. Sex: M ____ F ____

3. Marital status: a. Single___ b. Married____ c. Cohabiting d. Separated____ e. Divorced______

4. Education level (Highest attained):

a. None_____ b. Lower Primary____ c. Upper Primary ___ d. Secondary___ e. Post-Secondary___

5. Occupation: ______________________

6. Main language: ______________________________

***Introduction*** 

Hello. My name is _______________ and I am conducting a study on behalf of the Aga Khan Foundation. The Aga Khan Foundation has been working in [village] since [year]. You have been invited today because you have a specific position in the community, which we believe provides you with unique perspectives on our study topic: the social and cultural factors that influence dietary practices of mothers and their children. During this discussion, I want to focus on food preparation and consumption practices in the community, what influences these practices and especially how pregnant women and new mothers are involved with these practices, and why. We would also like to discuss any projects, from government or outside organizations, focused on these practices. Your opinions are very important to us and will help us to better understand how we can make a positive contribution to improving nutrition in this community.

We would like to record this discussion in order to remember the discussion and to present your views correctly in a report. No one outside the team here today will listen to this discussion and the recordings of this discussion will not mention you by name. Your name will not be shared with anyone. We will ensure that the information that you provide remains only with us. Is it okay if we record this discussion? [get informed consent on recording].

You should feel free to say whatever you think and feel. Your participation in this discussion is completely voluntary. You should not feel obligated to participate. There is no financial compensation for your participation, however we do hope that you will participate. If you are uncomfortable with a question, you can decide not to answer it. Do you have any questions about what I have said so far? [obtain response] [gain consent to proceed].

We thank you for giving us your time.

***Introductory questions*** 

*We would like to start by asking you a few introductory questions.* 

1. Let us start by talking about your role in the community. Can you describe your role to us?

*Probes:* How does your role influence community practices around nutrition? Who do you influence?

***For Community Elder:*** How many households are in your village? Approximately how many households are female-headed? What are the main languages spoken in your village? What are the main religious groups?

2. What influences the decisions that households in this community make around what food to eat?

*Probes:* Are these influences the same for most households? If not, what are the differences?

3. When you are told that a woman or child is “malnourished”, what do you think this means?

***For CHWs and leader of a Community Nutrition Group:*** Do you think women and children are malnourished in this community? If yes, why? If no, why not?

***Domain 1: Foods for various groups and consumption***

*We would now like to ask you about the types of foods commonly consumed by pregnant women, mothers, infants and children under 2 years of age.*

1. What foods are considered preferable for pregnant women in your community?

*Probes:* Why these foods? Are these foods consumed throughout the year? Do pregnant women of all ages consume these same foods?

2. What foods are considered preferable for breastfeeding women in your community?

*Probes:* Why these foods? Are these foods consumed throughout the year? Do breastfeeding women of all ages consume these same foods?

3. Can you describe the common feeding practices among mothers for babies below 6 months of age in your community?

*Probes:* Are babies this age exclusively breastfed? If not, why not?

What foods are babies below 6 months of age fed? Are these foods consumed throughout the year?

Why these foods? Are there differences in the feeding practices of mothers for babies below 6 months of age depending on whether the baby is male or female? If yes, what are these differences?

4. What foods are most commonly fed to children below 2 years in your community? Are these foods consumed throughout the year?

*Probes:* Why these foods? Are there differences in the feeding practices of mothers for children below 2 years of age depending on whether the child is male or female? If yes, what are these differences?

5. Can you tell us about how food is distributed and consumed within a typical household in your community?

*Probes:* Is food distributed equally among all household members? If not, who in the household is given priority access to food? Does this priority access to food change at different times of the year? If so, why?

Who decides who should eat what food and how much?

***Domain 2: Beliefs and practices influencing nutrition of mother and child***

*We will now ask a few questions relating to the community’s beliefs and social attitudes towards different foods and food practices, especially in relation to pregnant women, mothers and their children.*

1. Can you tell us about the social and cultural meanings attached to different foods in this community?

*Probes:* Which foods are seen to be most important for people’s health? Which foods are seen as signifying wealth or social status in the community? Do the social and cultural meanings attached to different foods change in times of economic difficulty? If so, how?

2. How do social attitudes around different food types differ for men and women? How do these attitudes differ for children, youth and adults?

3. What foods are seen to be most important for women to consume to ensure the birth of a healthy child?

*Probes:* Why those foods? What types of foods are considered taboo (bad for the health of mother and child or having a different type of negative social value) for pregnant women in this community? Do most people in the community share these beliefs? What is the origin of these beliefs?

4. What are the common perspectives around breastfeeding and weaning practices in the community?

*Probes:* What are beliefs around breastfeeding for babies less than 6 months of age? What are beliefs around breastfeeding for children over 6 months of age? What types of foods are considered taboo (bad for the health of mother and child or having a different type of negative social value) for breastfeeding women in this community? Do most people in the community share these beliefs? What is the origin of these beliefs?

5. What types of foods are considered taboo (bad for the health of mother and child or having a different type of negative social value) for children below 2 years of age in this community?

*Probes:* Do most people in the community share these beliefs? What is the origin of these beliefs?

***Domain 3: Economic factors influencing nutrition of mother and child***

*We will ask you a few questions relating to the different types of economic factors that influence access to food.*

1. What are the main economic challenges that people in this community must cope with?

*Probes:* Challenges related to production, sales/cash incomes, other factors?

2. Are all households affected in the same way by these economic challenges?

*Probes:* Why are some households more resilient to economic “shocks” than others?

3. What economic challenges affect access to food in this community?

*Probes:* Are all households affected equally by these challenges in the same way/with the same level of severity? If no, what are the differences?

***Domain 4: Gender issues influencing nutrition of mother and child***

*We now want to ask you a few questions on how the roles of men and women, both within the community and in the household, influence attitudes and behavior related to accessing and consuming food.*

1. In this community, who within the household typically decides what food to produce?

*Probes:* Who typically decides on what food to purchase? Who typically decides on what foods to consume?

2. How much influence do women have in determining the types of foods that they and their children will consume?

*Probes:* When pregnant, who (or what) influences women’s decisions on types of foods that they will consume? When breastfeeding, who (or what) influences women’s decisions on types of foods that they will consume? When with children less than 2 years of age, who (or what) influences women’s decisions on types of foods that they and their children will consume?

***Domain 5: Nutrition-focused interventions in Cabo Delgado***

*We will now ask you a few questions around the types of projects and/or messages around food that have been received in this community.*

1. Has the government or an outside organization introduced projects in your community around improving access to food?

*Probes:* Do you know who implemented these projects? What are (or were) these projects called? What are (or were) the objectives of these projects? Do you believe that these projects have been of value to your community? Why or why not?

2. Has the government or an outside organization introduced projects in your community focused on improving food consumption practices of pregnant women and mothers with children less than 2 years of age?

*Probes:* Do you know who implemented these projects? What are (or were) these projects called? What are (or were) the objectives of these projects? Do you believe that these projects have been of value to your community? Why or why not?

3. What types of messages do people in your community, especially women, receive around food?

*Probes:* On food preparation? On food storage? On food consumption? Are all these messages useful?

Why or why not?

4. Are you aware of any government directives focused on improving access to food in your community?

If yes, what are these directives?

*Probes:* How have these directives been applied in your community?

***Ending/closing questions***

1. Is there anything else you want to say about any other factors affecting nutrition and wellbeing of mothers and children in your community?

*Probes:* Add follow up question/s depending on what they choose to add.

***Thank participant now and offer to answer any final questions.***

### Appendix B.2. Question Guide for In-Depth Interviews (IDIs) with Pregnant Women, Breastfeeding Mothers and Women with Children under Two Years of Age

**Study:** Influence of socio-cultural, gender and economic factors on maternal and child nutrition practices in Cabo Delgado province, Mozambique

**Interviewer number:** _________

**Date:** __________________ **Venue:** _______________________

**Interviewer:** ___________________

**Site/District:** ___________________

**Participant category:** □ Pregnant woman

□ Breastfeeding mother with baby <6 mths

□ Woman with children under two years of age

***IDI participant characteristics*** [Collect these details for every IDI]

1. Age: ___________

2. Marital status: a. Single___ b. Married____ c. Cohabiting d. Separated____ e. Divorced______

3. Education level (Highest attained):

a. None_____ b. Lower Primary____ c. Upper Primary ___ d. Secondary___ e. Post-Secondary_

4. Occupation: ______________________

5. Main language: ______________________________

***Introduction***

Hello. My name is _______________ and I am conducting a study on behalf of the Aga Khan Foundation. The Aga Khan Foundation has been working in [village] since [year]. I want to hear about your opinions and experiences related to nutrition of mother and child in your communities. This research aims to shed light on the importance of social and cultural factors that influence diet practices of mothers and their children. During this discussion, I want to focus on food distribution and consumption practices in the community, what influences these practices and especially how pregnant women and new mothers are involved with these practices, and why. We would also like to discuss any projects, from government or outside organizations, focused on these practices. Your opinions are very important to us and will help us to better understand how we can make a positive contribution to improving nutrition in this community.

I would like to record this discussion in order to remember the discussion and to present your views correctly in a report. No one outside the team here today will listen to this discussion and the recordings of this discussion will not mention you by name. Your name will not be shared with anyone. We will ensure that the information that you provide remains only with us. Is it okay if we record this discussion? [get informed consent on recording].

You should feel free to say whatever you think and feel. Your participation in this discussion is completely voluntary. You should not feel obligated to participate. There is no financial compensation for your participation; however, we do hope that you will participate. If you are uncomfortable with a question, you can decide not to answer it. Do you have any questions about what I have said so far?

[Obtain response] [Gain consent to proceed].

We thank you for giving us your time.

***Introductory questions***

*I would like to start by asking you a few introductory questions.*

1. What influences the decisions that you make around what food to eat?

*Probes:* Are these influences the same for most women like you? If not, what are the differences?

2. When you are told that a woman or child is “malnourished”, what do you think this means?

*Probes:* What do you think makes a child “malnourished”?

***Domain 1: Foods for various groups and consumption***

*We would now like to ask you about the types of foods commonly consumed by pregnant women, mothers, infants and children under 2 years of age.*

1. What foods are considered preferable for pregnant women in your community?

*Probes:* Why these foods? Are these foods consumed throughout the year? Do pregnant women of all ages consume these same foods?

2. What foods are considered preferable for breastfeeding women in your community?

*Probes:* Why these foods? Are these foods consumed throughout the year? Do breastfeeding women of all ages consume these same foods?

3. Can you describe the common feeding practices among mothers for babies below 6 months of age in your community?

*Probes:* Are babies this age exclusively breastfed? If not, why not?

What foods are babies below 6 months of age fed? Are these foods consumed throughout the year?

Why these foods? Are there differences in the feeding practices of mothers for babies below 6 months of age depending on whether the baby is male or female? If yes, what are these differences?

4. What foods are most commonly fed to children below 2 years in your community? Are these foods consumed throughout the year?

*Probes:* Why these foods? Are there differences in the feeding practices of mothers for children below 2 years of age depending on whether the child is male or female? If yes, what are these differences?

5. Can you tell me how food is distributed and consumed within your household?

*Probes:* Is food distributed equally among all household members? If not, who in the household is given priority access to food? Does this priority access to food change at different times of the year? If so, why?

Who decides who should eat what food and how much?

***Domain 2: Beliefs and practices influencing nutrition of mother and child***

*I will now ask a few questions relating to the community’s beliefs and social attitudes towards different foods and food practices, especially in relation to pregnant women, mothers and their children.*

1. Can you tell me about the social and cultural meanings attached to different foods in your community?

*Probes:* Which foods are seen to be most important for people’s health? Which foods are seen as signifying wealth or social status in the community? Do the social and cultural meanings attached to different foods change in times of economic difficulty? If so, how?

2. How do social attitudes around different food types differ for men and women in your family? How do these attitudes differ for children, youth and adults?

3. What foods are seen to be most important for women to consume to ensure the birth of a healthy child?

*Probes:* Why those foods? What types of foods are considered taboo (bad for the health of mother and child or having a different type of negative social value) for pregnant women in this community? Do most people in the community share these beliefs? What is the origin of these beliefs?

4. What are the common perspectives around breastfeeding and weaning practices in the community?

*Probes:* What are beliefs around breastfeeding for babies less than 6 months of age? What are beliefs around breastfeeding for children over 6 months of age? What types of foods are considered taboo (bad for the health of mother and child or having a different type of negative social value) for breastfeeding?

Do most people in the community share these beliefs? What is the origin of these beliefs?

5. What types of foods are considered taboo (bad for the health of mother and child or having a different type of negative social value) for children below 2 years of age in this community?

*Probes:* Do most people in the community share these beliefs? What is the origin of these beliefs?

***Domain 3: Economic factors influencing nutrition of mother and child***

*I will now ask you a few questions relating to the different types of economic factors that influence access to food.*

1. What are the main economic challenges that you and your household cope with?

*Probes:* Challenges related to production, sales/cash incomes, other factors?

2. Are all households affected in the same way by these economic challenges?

*Probes:* Why are some households more resilient to economic “shocks” than others?

3. What economic challenges affect your access to food?

***Domain 4: Gender issues influencing nutrition of mother and child***

*Now I want to ask you a few questions on how the roles of men and women, both within the community and in the household, influence attitudes and behavior related to accessing and consuming food.*

1. In this community, who within the household typically decides what food to produce?

*Probes:* Who typically decides on what food to purchase? Who typically decides on what foods to consume?

2. How much influence do women have in determining the types of foods that they and their children will consume?

*Probes:* When pregnant, who (or what) influences your decision on types of foods that you consume?

When breastfeeding, who (or what) influences your decisions on types of foods that you consume?

When taking care of children less than 2 years of age, who (or what) influences your decisions on types of foods that your children will consume?

***Domain 5: Nutrition-focused interventions Cabo Delgado***

*I will now ask you a few questions around the types of projects and/or messages around food that you may have received.*

1. Has the government or an outside organization introduced projects in your community around improving access to food?

*Probes:* If yes, do you know who implemented these projects? What are (or were) these projects called?

What are (or were) the objectives of these projects? Do you believe that these projects have been of value to you? Why or why not?

2. Has the government or an outside organization introduced projects in your community focused on improving food consumption practices of pregnant women and mothers with children less than 2 years of age?

*Probes:* If yes, do you know who implemented these projects? What are (or were) these projects called?

What are (or were) the objectives of these projects? Do you believe that these projects have been of value to you? Why or why not?

3. What types of messages do people in your community, especially women, receive around food?

*Probes:* On food preparation? On food storage? On food consumption? Are all these messages useful to you? Why or why not?

4. Are you aware of any government directives focused on improving access to food in your community?

If yes, what are these directives?

*Probes:* How have these directives been applied in your community?

***Ending/closing questions***

1. Is there anything else you want to say about any other factors affecting nutrition of mothers and child in your communities?

*Probes:* Add follow up question/s depending on what they choose to add.

***Thank participant now and offer to answer any final questions.***
